# A New Orbivirus Isolated from Mosquitoes in North-Western Australia Shows Antigenic and Genetic Similarity to Corriparta Virus but Does Not Replicate in Vertebrate Cells

**DOI:** 10.3390/v8050141

**Published:** 2016-05-20

**Authors:** Jessica J. Harrison, David Warrilow, Breeanna J. McLean, Daniel Watterson, Caitlin A. O’Brien, Agathe M.G. Colmant, Cheryl A. Johansen, Ross T. Barnard, Sonja Hall-Mendelin, Steven S. Davis, Roy A. Hall, Jody Hobson-Peters

**Affiliations:** 1 Australian Infectious Diseases Research Centre, School of Chemistry and Molecular Biosciences, The University of Queensland, St Lucia 4072, Australia; jessica.harrison@uqconnect.edu.au (J.J.H.); breeanna.mclean@uqconnect.edu.au (B.J.M.); d.watterson@uq.edu.au (D.W.); caitlin.obrien@uqconnect.edu.au (C.A.O.B.); a.colmant@uq.edu.au (A.M.G.C.); rossbarnard@uq.edu.au (R.T.B.); roy.hall@uq.edu.au (R.A.H.); 2 Public Health Virology Laboratory, Department of Health, Queensland Government, P.O. Box 594, Archerfield 4108, Australia; David.Warrilow@health.qld.gov.au (D.W.); Sonja.Hall-Mendelin@health.qld.gov.au (S.H.-M.); 3 School of Pathology and Laboratory Medicine, The University of Western Australia, Nedlands 6009, Australia; cheryl.johansen@uwa.edu.au; 4 Berrimah Veterinary Laboratory, Department of Primary Industries and Fisheries, Darwin 0828, Australia; steven.davis@nt.gov.au

**Keywords:** insect-specific virus, reovirus, orbivirus, mosquito, arbovirus, virus discovery, *Culex annulirostris*, double-stranded RNA

## Abstract

The discovery and characterisation of new mosquito-borne viruses provides valuable information on the biodiversity of vector-borne viruses and important insights into their evolution. In this study, a broad-spectrum virus screening system, based on the detection of long double-stranded RNA in inoculated cell cultures, was used to investigate the presence of novel viruses in mosquito populations of northern Australia. We detected and isolated a new virus (tentatively named Parry’s Lagoon virus, PLV) from *Culex annulirostris, Culex pullus, Mansonia uniformis* and *Aedes normanensis* mosquitoes that shares genomic sequence similarities to Corriparta virus (CORV), a member of the *Orbivirus* genus of the family *Reoviridae*. Despite moderate to high (72.2% to 92.2%) amino acid identity across all proteins when compared to CORV, and demonstration of antigenic relatedness, PLV did not replicate in several vertebrate cell lines that were permissive to CORV. This striking phenotypic difference suggests that PLV has evolved to have a very restricted host range, indicative of a mosquito-only life cycle.

## 1. Introduction

Viruses of the *Reoviridae* family are icosahedral, non-enveloped and consist of a 9–12 segmented double-stranded RNA (dsRNA) genome [1]. The family consists of two clear subfamilies, the *Spinareovirinae* (“spiked” core particles) and the *Sedoreovirinae* (“spikes” absent on core particles). Reoviruses are separated into 16 distinct genera [1]. In addition to infecting plants, reoviruses are pathogens of a wide variety of vertebrates and invertebrates, including crustaceans, fish, insects, reptiles and mammals [1].

The genus *Orbivirus* (subfamily: *Sedoreovirinae*), characterised by the presence of a 10-segment RNA genome, is the largest of the *Reoviridae* genera and contains 22 distinct virus species [1]. Bluetongue virus, African horse sickness virus and epizootic haemorrhagic disease virus are regarded as the most economically important viruses within this genus, due to direct economic losses associated with herd morbidity and mortality as well as a significant loss through restriction on animal movement [2].

Sequence analysis of the highly conserved major subcore structural protein (T2) is commonly used to assign novel isolates to already recognized orbivirus species, or to support the designation of a new species. Typically, a distinct viral species within this genus will have at least 74% nucleotide identity in the gene encoding T2 [1]. There are two distinct vector-associated groups of orbiviruses based on this gene. Those viruses in which segment 3 (VP3) encodes T2 are associated with culicoid midges. In contrast, T2 is encoded by segment 2 (VP2) by those viruses that are mosquito- or tick-borne.

In northern Australia, orbiviruses are highly prevalent [3,4,5,6]. In addition to the midge-transmitted and agriculturally significant bluetongue virus, a number of mosquito-borne orbiviruses are also detected in this region [4,6,7,8]. Middle Point orbivirus and Stretch Lagoon orbivirus are often isolated during mosquito or animal sera screening. Extensive serological studies have identified neutralizing antibodies to Middle Point and Stretch Lagoon orbiviruses in a number of vertebrate species, including, cattle, horses, donkeys and goats [4,7,9,10]. Despite the fact that no overt disease has been attributed to infection with these viruses, the high prevalence of neutralizing antibodies in a range of vertebrate species flags the importance of continual arboviral surveillance.

Corriparta virus (CORV) was first isolated from *Culex annulirostris* mosquitoes collected near Mitchell River, North Queensland, Australia in 1960. In addition to the prototype species, CORV-MRM1, the International Committee on Taxonomy of Viruses (ICTV) recognizes an additional five serotypes/strains of CORV: Acado virus, CSIRO109, V654, V370 and Jacareacanga viruses [1]. The different serotypes/strains have been identified in Australia, Africa and South America via traditional serological methods. The identification of CORV-neutralizing antibodies in a wide range of vertebrate species, including wild and domestic birds, cattle, marsupials, horses and man [5] is indicative of a wide host range, despite the fact that no overt disease due to infection has been observed. Despite the isolation of the prototype CORV in 1960, the genome sequence of this virus has only recently been elucidated [11]. Following the availability of the CORV genome, an additional member was assigned to the CORV species, this being California Mosquito Pool virus (CMPV) [11].

During studies to determine the prevalence and biodiversity of mosquito-borne viruses in northern Australia, an unknown RNA virus was detected in *Culex annulirostris* samples via the presence of long double-stranded RNA in inoculated mosquito cells [12]. Here, we describe the isolation and characterisation of the new virus, tentatively named Parry’s Lagoon virus (PLV), after the region in which the mosquitoes were collected that yielded the prototype virus. The proposed virus failed to replicate in cells of vertebrate origin, despite its close genetic and antigenic relatedness to the vertebrate-infecting CORV and may represent the first isolation of an insect-specific member of the *Orbivirus* genus in Australia.

## 2. Materials and Methods

### 2.1. Cell Culture

C6/36 *(Aedes albopictus*) cells were maintained at 28 °C in Rosewell Park Memorial Institute 1640 (RPMI) media and supplemented with 5% fetal bovine serum (FBS). All vertebrate cells used in this study were maintained at 37 °C 5% CO_2_. BHK (baby hamster kidney), and Vero African green monkey kidney) cells were maintained in Dulbecco’s Modified Eagle Medium (DMEM) containing 5% FBS, while DF-1 (chicken embryo fibroblast) cells (provided by Dr. David Williams, CSIRO) were maintained in DMEM containing 10% FBS. All media was supplemented with 50 U/mL penicillin, 50 μg/mL streptomycin and 2 mmol/L l-glutamine.

### 2.2. Mosquito Collection and Initial Pool Screening

The study sites described in this paper have previously been described in detail [13,14]. Adult mosquitoes were collected in the Kimberley region of northern Western Australia [15] using methods already described [16]. Mosquitoes were homogenised [17] in pools of up to 25 mosquitoes. Homogenates were inoculated onto 96 well monolayers of C6/36 cells and subsequently passaged onto additional 96 well monolayers of C6/36, Vero and PS-EK (porcine stable-equine kidney) cells as described elsewhere [18]. Monolayers were examined microscopically for evidence of infection, and C6/36 cell monolayers were fixed and assayed by fixed cell ELISA using a panel of *flavivirus* and *alphavirus* generic and specific monoclonal antibodies [19].

### 2.3. Parry’s Lagoon Virus Detection and Isolation from Mosquito Homogenates

Mosquito pools that were negative for flaviviruses and alphaviruses and did not induce cytopathic effect when inoculated onto vertebrate cell lines (see Section 2.2) were further assessed for the presence of novel viruses using the MAVRIC system developed in our lab [12]. Briefly, virus isolation was performed using mosquito homogenate inoculation onto C6/36 monolayers and incubation at 28 °C for 5–7 days. The culture supernatant was collected and stored at −80 °C, while the cell monolayer was fixed with 20% (*v*/*v*) acetone, 0.2% (*w*/*v*) BSA in PBS (phosphate buffered saline). For subsequent ELISA analysis, the plates were blocked with 150 μL per well ELISA blocking buffer (0.05 M Tris/HCl (pH 8.0), 1 mM EDTA, 0.15 M NaCl, 0.05% (*v*/*v*) Tween 20, 0.2% *w*/*v* casein) for 1 h at room temperature before probing with 50 μL/well mAbs 3G1 and 2G4 (anti-dsRNA) diluted in blocking buffer. Following a 1 h incubation at 37 °C, the plates were washed four times with PBS containing 0.05% tween-20 (PBST). HRP-conjugated goat anti-mouse Ig (DAKO) was diluted 1/2000 in blocking buffer (50 μL/well), was added and incubated at 37 °C for 1 h, prior to washing six times with PBST. Finally, 100 μL/well substrate solution [1 mM 2,2-azino-bis(3-ethylbenzthiazoline-6-sulfonic acid) (ABTS), 3 mM H_2_O_2_ in a buffer prepared by mixing 0.1 M citric acid with 0.2 M Na_2_HPO_4_ to give a pH of 4.2] was added per well and plates were incubated in the dark at room temperature for 1 h. Absorbance was measured at 405 nm.

Samples that tested positive by the MAVRIC ELISA were further analysed. For detection of PLV sequence, RNA was extracted from 150 μL of culture supernatant using a Macherey-Nagel Nucleospin Viral RNA isolation kit as per the manufacturer’s instructions (Macherey-Nagel, Bethlehem, PA, USA). Reverse-transcription PCR (RT-PCR) was performed using the primer pair of CORV_like_F (5′-TTATCGGCAGACGGGATTCG) and CORV_like_R (5′-CGCTTTCGTTAGCACCATCG) and an Invitrogen Superscript III One-step RT-PCR system with Platinum *Taq* DNA polymerase. Cycling conditions were: [45 °C/30 min]_1_; [98 °C/2 min]_1_; [94 °C/30 s | 45 °C/30 s | 68 °C/30 s]_40_; [68 °C/10 min]_1_. Amplicons were purified by agarose gel electrophoresis and extracted using a Gel Extract II PCR Clean-up Kit (Macherey-Nagel, Bethlehem, PA, USA). Sequencing was performed by the Australian Genome Research Facility (AGRF, Brisbane, Australia).

### 2.4. Virus Culture

Virus stocks were generated by infecting C6/36 monolayers with virus at a multiplicity of infection (MOI) of 0.1. After incubation at 28 °C for 2 h, inoculum was removed and replaced with fresh growth media containing 2% FBS. Supernatant was harvested at 5 dpi and centrifuged at 3000 rpm, 4 °C for 10 min. Clarified supernatant was passed through a 0.45 μM filter, supplemented with additional FBS to increase total concentration to 10% and stored at −80 °C. Virus stock titers were calculated based on the 50% tissue culture infectious dose [20].

### 2.5. Electron Microscopy

Virions were harvested and concentrated via polyethylene glycol precipitation. Following overnight incubation, the resulting pellet was resuspended in TNE buffer (120 mM NaCl, 30 mM H_3_BO_3_, 1% Triton X-100, 0.1% SDS, 5 mM EDTA, pH: 9.0). Virus-infected supernatant was then underlayed with a 20% sucrose cushion before ultracentrifugation at 28,000 rpm for 2 h at 4 °C. The purified virions were harvested and buffer exchanged into PBS prior to loading onto hydrophilic copper grids and negatively stained with a 1% uranyl acetate solution. Grids were viewed using a F30 transmission electron microscope.

### 2.6. Genome Sequencing and Phylogenetic Analysis

Viral RNA was prepared for sequencing by infecting a monolayer of C6/36 cells at a MOI of 0.1. After incubating at 28 °C for 2 h, inoculum was removed and replaced with fresh growth media containing 2% FBS. Virus was harvested 6 dpi by centrifuging supernatant at 3000 rpm, 4 °C for 10 min before passing it through a 0.45 μM filter. Virus-infected supernatant was then mixed in a 1:4 ratio of 40% polyethylene glycol 8000 (PEG 8000) and incubated on a mixing wheel with slow rotation overnight at 4 °C. The concentrated virions were then pelleted by centrifuging at 10,000× *g*, 4 °C for 1 h before resuspending the resulting pellet in sterile PBS. RNA was then extracted using a Macherey-Nagel Nucleospin Viral RNA isolation kit as per the manufacturer’s instructions, with the omission of carrier RNA from the lysis buffer. Next generation sequencing was performed by the AGRF (Brisbane, Australia) using the Illumina HiSeq2000 platform. Reads were assembled using Geneious R8 (8.0.5) software and the CORV MRMI isolate genome as a reference (Accession numbers: KC853042-KC853051). PLV open reading frames (ORFs) were identified using the NCBI ORF finder (http://www.ncbi.nlm.nih.gov/gorf/gorf.html) and the ORF sequences deposited in Genbank under accession numbers KU724110-KU724119.

Phylogenetic analysis was performed on highly conserved genes encoding VP1 (Pol), VP2 (T2) and VP7 (T13). Nucleotide sequences of the ORFs were aligned with Geneious R8 (v 8.0.5) using the MUSCLE algorithm on 8 iterations, a distance measure of kmer4_6 for the first iteration, and a distance measure of pctid_kimura for subsequent iterations [21]. Phylogenetic trees based on the alignments were constructed in MEGA-7.0.14 [22] using maximum likelihood, a General Time Reversible substitution model, a gamma distribution (5 discrete gamma categories) and invariant rates among sites (determined to be optimal using jModelTest-2.1.6 software [23,24] minimizing deltaAIC). Bootstrap analysis was performed with 1000 replicates, and the trees were rooted using the divergent orbivirus St. Croix River virus (SCRV) as an outgroup.

### 2.7. Serological Cross-Reactivity Studies

IFA analysis was performed using two rabbit antisera generated against CORV (serum B97 raised to an unknown CORV strain and R167 raised to CORV strain CSIRO109) was performed to assess serological cross-reactivity to PLV. Monolayers of C6/36 cells were grown on glass coverslips before inoculating with PLV, CORV or mock-infected at an MOI of 1. Coverslips were cultured for 4 dpi, before fixing in a solution containing 4% formaldehyde and 0.1% triton X-100 and probed using CORV antiserum, or negative rabbit serum (diluted 1/100), using methods previously detailed [25]. RT-PCR was used to confirm that cross-contamination of CORV in PLV samples had not occurred.

### 2.8. Microneutralisation Assay

Microneutralisation assays were performed to assess if antisera raised against CORV (refer to section 2.7) are able to neutralize PLV. Assays were performed as previously detailed [26] utilizing C6/36 cells, titrating the rabbit serum from a 1/10 dilution and using 450–1000 infectious particles, as determined by back-titration. The neutralization titer was taken as the highest dilution that inhibited all CPE.

### 2.9. Vertebrate Cell Infection Assays

IFA was performed with anti-dsRNA mAb, 3G1, to assess the permissiveness of vertebrate cell lines to infection with PLV. Monolayers of C6/36, BHK and Vero cells were grown on glass coverslips before inoculating with PLV, CORV or mock-infected at an MOI of 1 and were cultured for 3 and 9 dpi. Coverslips were fixed in a PBS solution containing 4% formaldehyde and 0.1% Triton-X 100 before being probed with 3G1 as described [12].

## 3. Results

### 3.1. Detection, Isolation and Culture of the Prototype PLV Isolate

**Figure 1:**
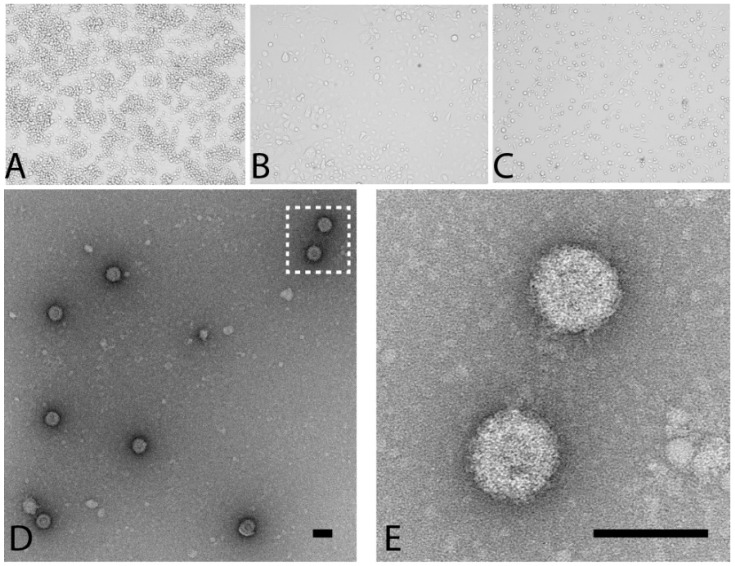
Parry’s Lagoon virus (PLV) Morphology and Growth Characteristics. PLV induces distinct CPE on C6/36 monolayers at 5 days post infection. (**A**) mock-infected C6/36 cells; (**B**) PLV-infected C6/36 cells and (**C**) Corriparta virus (CORV)-infected C6/36 cells; (**D**,**E**) Culture supernatant of C6/36 cells infected with PLV was concentrated and virions negatively stained using a 1% uranyl acetate solution. Scale bar represents 80 nm.

As part of routine surveillance for arboviruses, mosquitoes were collected in the northern regions of Western Australia in 2010. A subset of *Culex annulirostris* mosquito pools that were negative for flaviviruses and alphaviruses and did not cause cytopathic effect (CPE) in vertebrate cells (*n* = 138) were examined for the presence of insect-specific viruses. The pools were screened using a novel ELISA-based system developed in our laboratory for the detection of viral replication in cell culture (named MAVRIC, [12]). Of the 7 pools that were positive using this system, three (numbers K71435, K71497 and K71551; Table 1) displayed significant CPE five days post infection of C6/36 cell monolayers, causing cell monolayer disturbance, vacuolation and loss of uniformity in size and shape of cells (Figure 1B). An initial round of Ion Torrent sequencing of one isolate (K71551) identified the virus as an orbivirus (*Reoviridae* family). The short sequence obtained (approximately 850 bp) was of the gene encoding the viral helicase and NS4 proteins and shared a 77% nucleotide and 68% amino acid identity to the prototype isolate of Corriparta virus, strain MRM1 (CORV-MRM1). The new virus was tentatively named Parry’s Lagoon virus (PLV), after the region in which the prototype was first identified.

### 3.2. PLV Virions Display Typical Reovirus Morphology

When purified PLV virions were examined by transmission electron microscopy, small, icosahedral non-enveloped particles were observed (Figure 1D,E). This morphology is consistent to that described for other members of the *Reoviridae* [9,27,28]. However, with an average diameter of 79 nm, the virions are substantially larger than the 60 nm described for CORV [29], but within the size range generally accepted for orbiviruses (60–80 nm) [1] and may be attributed to the different fixation and staining methods used.

### 3.3. Detection of Other PLV Isolates from Mosquitoes Collected in Western Australia

RT-PCR screening of the other MAVRIC-positive pools from the Parry’s Creek 2010 cohort using PLV-specific primers, yielded a total of six PLV isolates (Table 1). Testing of an additional 15 mosquito pools collected from other locations within close proximity to Parry’s Creek, including Kununurra (approximately 80 km south-east) and Billiluna (approximately 500 km south) in 2011 that caused extensive CPE upon inoculation onto C6/36 cells and were positive by the MAVRIC ELISA, returned a further eight PLV isolates. In addition to *Culex annulirostris*, PLV isolates were also obtained from *Culex pullus, Mansonia uniformis* and *Aedes normanensis* mosquito species. Sequencing of a 399-nucleotide region of the helicase segment (seg-9, VP6) of each isolate gave a ≥97.5% nucleotide identity (Table 1) to the prototype, suggesting that they are likely to be strains of the same virus species.

It was noted that while pool K75749 was positive for PLV, it also induced CPE at passage 3 upon inoculation onto PS-EK and Vero cells. As no other PLV-positive pools induced CPE in vertebrate cells and sequencing of the 399 bp of segment 9 confirmed the presence of PLV, it is likely that an additional virus is present in that pool. RT-PCR using specific primers to two other reoviruses commonly detected in mosquito pools (Stretch Lagoon virus and Liao ning virus) failed to amplify a product. While this pool was negative for flaviviruses and alphaviruses during the initial isolation using immunological methods, these data were confirmed using *flavivirus* and *alphavirus* generic primer sets in RT-PCR.

### 3.4. Genome Sequence, Organisation and Phylogenetics

Next generation sequencing was performed on PLV RNA using the Illumina HiSeq 2000 platform and assembled using the CORV-MRM1 genome as a reference sequence. Partial sequence was obtained from 10 genome segments, consistent with the number of segments of an orbivirus genome (Accession numbers KU724110-KU724119). Complete sequence of each ORF was elucidated and further analysis of these ORFs revealed that 8 of the 10 segments (seg-1 to seg-8) consisted of a single ORF while the remaining two segments (seg-9 and seg-10) consisted of two overlapping ORFs, encoding VP6/NS4 and NS3/NS3a respectively, consistent with other orbiviruses. Pairwise alignments between the ORFs of each segment of the genomes of CORV and PLV demonstrated nucleotide identities ranging between 74.7% (VP3; OC1) and 81.2% (NS3/NS3a) and amino acid identities of 72.6% (VP6/NS4) and 95.3% (VP5; OC2) (Table 2).

**Figure 2:**
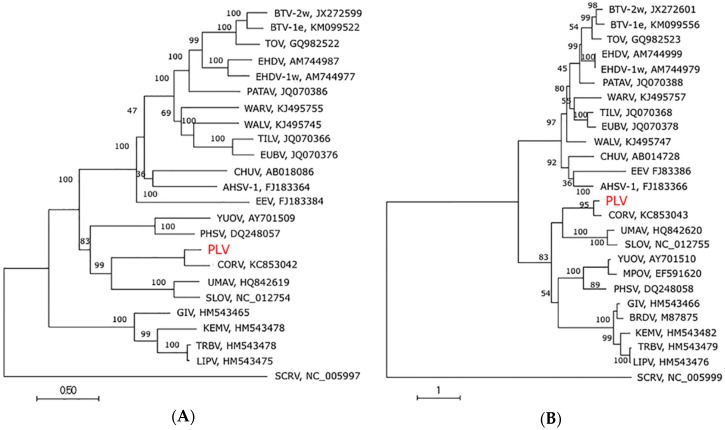

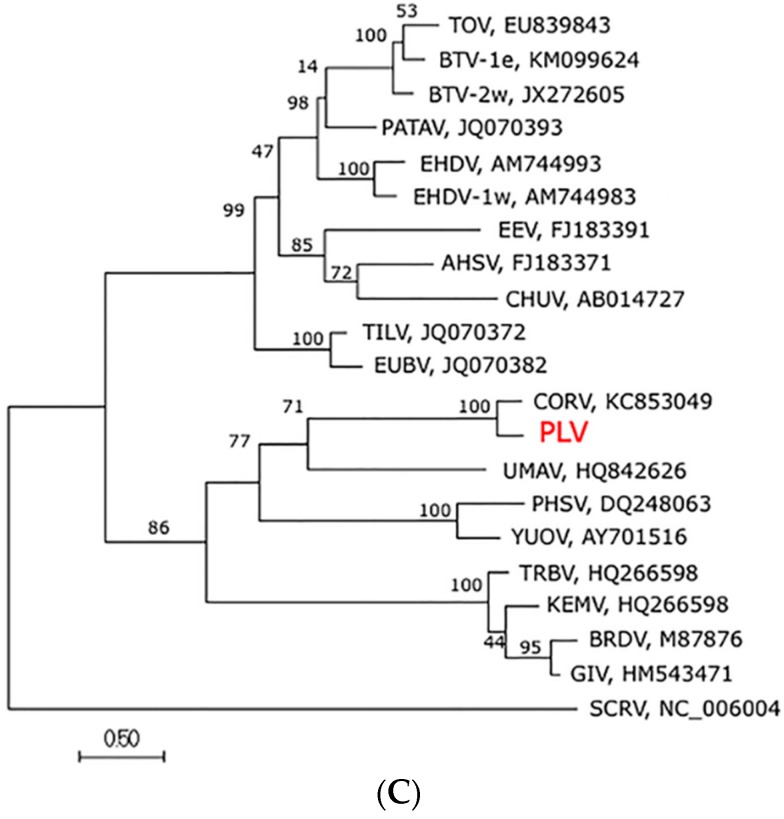
Phylogenetic analysis of *orbivirus* highly conserved genes. Maximum likelihood trees of highly conserved genes (**A**) VP1 (Pol); (**B**) T2 (subcore shell) and (**C**) T13 (outer core). Trees were constructed using a general time reversible model with gamma distribution and invariant sites (as determined by a jModelTest) with 1000 replicates. “e” and “w” denotes eastern and western isolates respectively. Accession numbers are denoted next to each virus. PLV accession numbers: KU724110-KU724119.

Phylogenetic trees were constructed using ORFs available for VP1, T2 and T13 proteins. In each case PLV clustered with CORV within the mosquito-associated orbivirus clade (Figure 2), with strong bootstrap support (>95). A separate phylogenetic tree prepared for the partial sequence of T13 was also supportive of inclusion of PLV within the mosquito-associated clade (Figure S1). PLV and CORV clustered together indicating a closer genetic relationship between these viruses, which was consistent with their higher amino acid identity (Table 3). An amino acid identity for T2 (subcore) of <91% has previously been proposed as a possible value for demarcation of species within the *Orbivirus* genus [30]. Based on this parameter, a 94.3% and 92% amino acid identity with CORV, respectively, would suggest that both PLV and CMPV are strains of the virus species CORV, as has been previously suggested [11].

### 3.5. PLV Does Not Replicate in Vertebrate Cells

**Figure 3:**
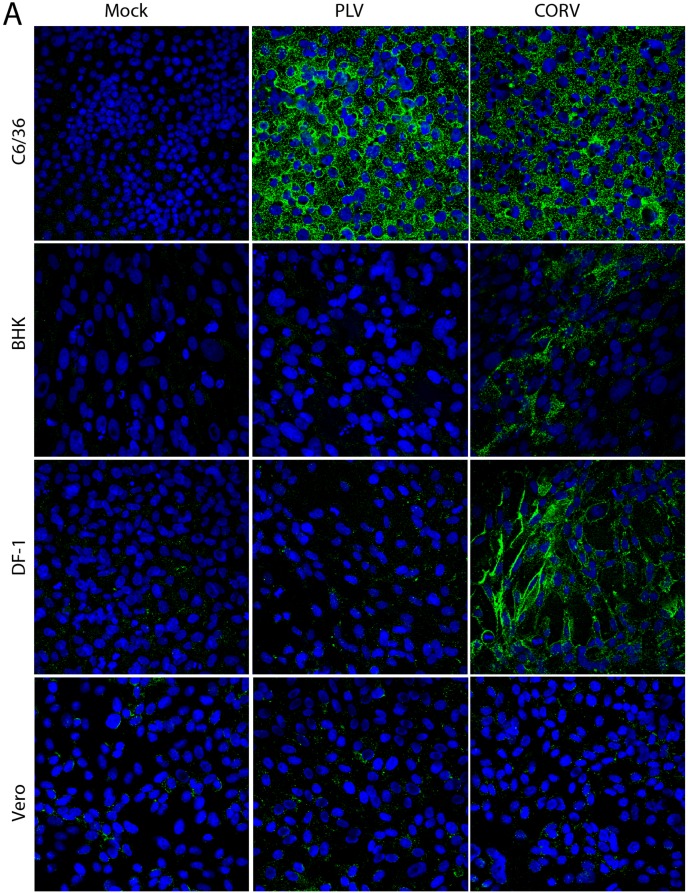

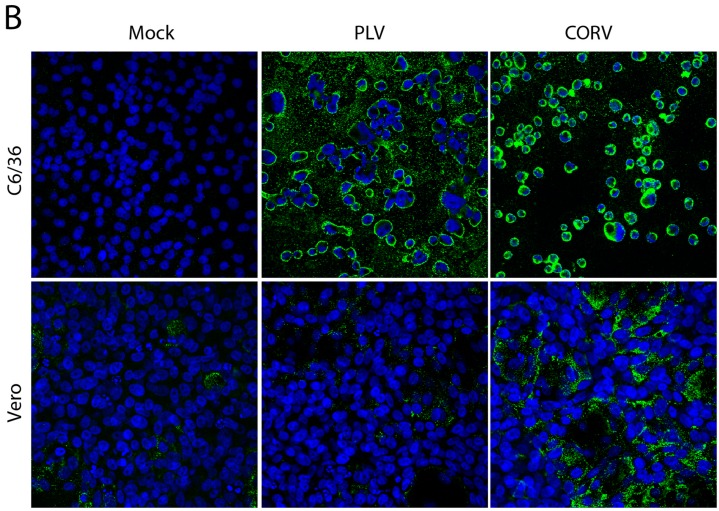
Vertebrate Cell Infection Assay. Mosquito (C6/36) and vertebrate (BHK, DF-1, Vero) cell monolayers were inoculated with PLV or CORV at an MOI of 1, or mock-infected and fixed 3 days post infect (**A**) or 9 days post infection (**B**). IFA analysis was performed with anti-dsRNA mAb 3G1 to detect replicating virus via dsRNA products [12]. Cell nuclei were stained with Hoechst 33342. Images were taken at ×40 magnification.

No CPE was observed in PS-EK cells at the third passage during initial isolations of PLV (Table 1). This contrasted with earlier studies of CORV that indicated it grows readily in porcine cells and causes CPE [29]. To investigate further, other cells of vertebrate origin were assessed for permissiveness to PLV (isolate K71551) and CORV (MRM1) by infecting monolayers of each cell line at an MOI of 1 and fixing after 3 days. IFA analysis was performed using anti-dsRNA mAbs to detect viral genomic and replicative dsRNA in the cytoplasm. We have previously reported the detection of the replicative dsRNA of other reoviruses such as Bluetongue virus and Liao ning virus using this protocol [12]. While PLV replication was clearly detected by the anti-dsRNA mAbs in C6/36 cells, there was no staining of BHK, DF-1 or Vero cells (Figure 3A) and no CPE observed. In contrast, CORV replicated in both C6/36, BHK and DF-1 cells as indicated by clear anti-dsRNA mAb-binding in IFA and by marked CPE (in the C6/36 and BHK cells) (Figure 3A). Consistent with previous reports, the replication of CORV was not detected in Vero cells until 9 dpi (Figure 3B). Cultures of Vero cells inoculated with PLV in parallel were negative by IFA under the same culturing conditions. Together, these data suggest that PLV displays a tropism that is substantially different to CORV and is likely to be restricted to insect cells.

### 3.6. Corriparta Antiserum Cross-Reacts with and Neutralizes Parry’s Lagoon Virus

**Figure 4:**
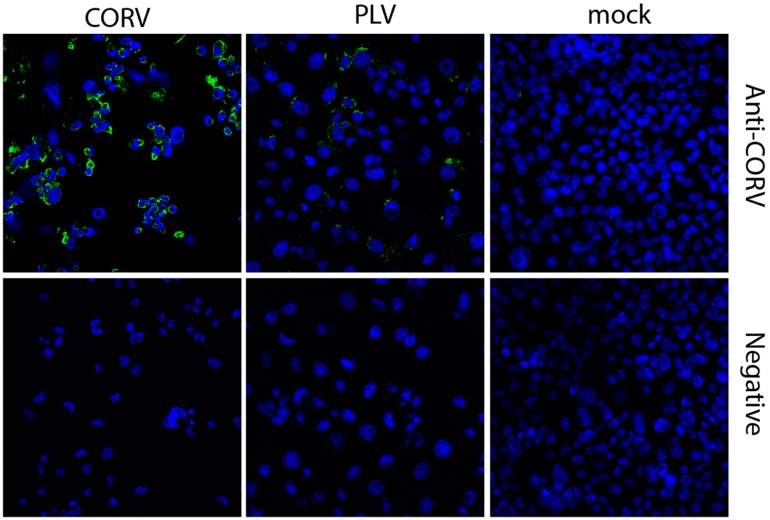
Serological Cross-Reactively Studies. C6/36 cells were inoculated with CORV or PLV at an MOI of 1, or mock-infected and fixed with 4% formaldehyde containing 0.1% triton X-100 after 4 days. IFA was performed by probing with rabbit sera raised against CORV CSIRO109l or naïve rabbit serum (negative) to assess cross-reactivity with PLV. Images were taken using ×40 magnification.

In order to determine if PLV is antigenically related to CORV, serological cross-reactivity studies and microneutralization assays were performed. Cross-reaction of CORV antiserum with PLV-infected cells in IFA confirmed that PLV shares similar antigenic structure to CORV, although the staining for PLV was not as intense as that for CORV (Figure 4). Similarly, two rabbit antisera produced to different strains of CORV also neutralized PLV-infection of C6/36 cell monolayers, but to different extents (Table 4). A four-fold difference between neutralizing titers of CORV and PLV by antiserum B97 was observed (80 *vs.* 20 respectively) indicating superior neutralization of CORV by this antiserum. However, when the second antiserum produced to the CORV CSIRO109 strain was assessed, this antiserum neutralized PLV at dilutions of at least 4-fold greater than CORV (PLV >1280 *vs.* CORV 320) and may suggest subtle antigenic differences even between strains previously assigned to the CORV species. Since no genome sequencing of CORV CSIRO109 is available to elucidate its genetic relatedness to PLV, it must be highlighted that CORV CSIRO109 was isolated using BHK [6] cells and thus does not display restricted host range. As expected, a negative control rabbit antiserum did not neutralize either virus.

## 4. Discussion

The *Orbivirus* genus is the largest of the *Reoviridae* family. These viruses are able to infect a wide range of arthropod and vertebrate species. During an investigation to determine the prevalence and biodiversity of insect-specific viruses in mosquitoes of northern Western Australia, a novel orbivirus was identified in pools of *Culex annulirostris*, *Culex pullus*, *Mansonia uniformis* and *Aedes normanensis* mosquitoes collected in 2010 and 2011 that had previously been screened for vertebrate-infecting viruses. PLV was genetically closely related to Corriparta virus (CORV), a virus that has also been isolated from mosquitoes in this region [8] and from mosquitoes and birds in other regions of northern Australia [31]. Despite high amino acid sequence identities over the polymerase, capsid and core proteins (86% (Outer capsid protein 1) to 95% (Outer capsid protein 2)) between PLV and CORV, PLV did not replicate in Vero, BHK or DF-1 cells. This contrasts with the efficient replication of CORV in each of these cell lines.

An amino acid identity for T2 of <91% has previously been proposed as a possible value for demarcation of species within the *Orbivirus* genus [30]. If this criterion alone is taken into consideration, a 94.3% amino acid identity for the T2 inner core protein between PLV and CORV-MRM1 would suggest that PLV should be included in the CORV species. Traditionally, orbiviruses were classified based on complement-fixation tests, group-specific ELISAs or agar-gel-immuno-diffusion tests [1,32]. Using these serology-based methods, viruses designated to the Corriparta serogroup include other viruses similarly isolated from *Culex* mosquito species, including CORV-MRM1 (Australia), Acado virus (Ethiopia) and Jacareacanga virus (Brazil). Isolates have also been identified in *Aedeomyia catastica* mosquitoes captured from the Ord River region of northern Western Australia (approximately 70 km from the Parry’s Creek region) between 1972 and 1976 [8]. CMPV was similarly isolated from *Culex* mosquitoes (*Culex tarsalis*) in North America, but has been proposed to become a member of the CORV species based on genetic sequence analysis [11]. Indeed, our own serological assessment of PLV with CORV antiserum confirms that PLV is antigenically related to CORV, but with neutralization values of 4 fold or greater between the two viruses. The current lack of full-genome sequencing data for other CORV serotypes apart from the prototype strain (and now for PLV) precludes accurate identification and classification of new serotypes as part of the CORV species or serogroup. Furthermore, with the ability of segmented viruses to reassort, taxonomic demarcation based purely on one segment may be insufficient. Indeed, studies have demonstrated extensive reassortment within the CORV serocomplex members [33]. To ensure more accurate taxonomic classification of PLV, future studies will also focus on the elucidation of the conserved terminal nucleotides of each genome segment, as these termini are often conserved within a single species [1].

Primary isolations of CORV were historically performed through inoculation of mosquito homogenate in suckling mouse brain [31]. However, with the advent of cell-culture based systems, more recent CORV isolates were identified through the inoculation of mosquito homogenate onto BHK or BSR cell monolayers and subsequent visualization of CPE [6,34]. In this context, the inability of PLV to replicate in vertebrate cells precluded its discovery using standard surveillance methods and highlights the utility of virus detection through the use of monoclonal antibodies to dsRNA [12].

While Australian CORV isolates have been derived from mosquitoes of both *Culex* and *Aedeomyia* genera, considering the proposed insect-specific tropism of PLV, it was interesting that PLV was also isolated from multiple mosquito genera. Given that vertical transmission has either been proposed or demonstrated for other insect-specific viruses [35,36,37], the presence of a virus in multiple mosquito genera is not consistent with mosquito-specific tropism. However, the isolation of other species of insect-specific viruses from mosquitoes of various genera has also been reported for viruses of other virus families, such as the *Bunyaviridae* [38,39]. Further studies assessing the growth of PLV in a broader range of vertebrate cell lines and mice will also assist in determining whether cryptic vertebrate hosts could be involved in the maintenance and transmission of PLV between mosquito species. Indeed, other reoviruses such as Yunnan orbivirus, Eyach virus, Cimodo virus, Fako virus and *Aedes psuedoscutellaris* reovirus similarly did not replicate in vertebrate cells *in vitro* during initial investigations, including Vero and BHKs [27,28,40,41,42]. However, both Yunnan orbivirus and Eyach virus were capable of infecting mice (10 week old intraperitoneal inoculation and suckling mice intracranial inoculation respectively) [27,41]. Members of the *Reoviridae* family also participate in a transmission cycle between insect hosts such as planthoppers or leafhoppers and the plants on which these insects feed on [43]. Hence, the ability of PLV to replicate in plants should also be considered, especially since mosquitoes feed on the nectar of plants as a sugar source. It is likewise plausible that the virus may be transmitted in the aquatic environment during the larval feeding stage. In any case, further investigations should be carried out to elucidate the mechanisms for host range restriction and persistence of PLV in nature.

The striking difference in cell tropism between PLV and CORV, despite the close genetic relatedness was unexpected. However, dramatic shifts in host cell tropism between closely related mosquito-borne viruses has been observed previously. A notable example is the lack of growth of Rabensburg virus (RaBV—a tentative lineage of the West Nile species complex) in vertebrate cells at 37 °C [44,45]. While other West Nile virus strains replicate efficiently in a range of vertebrate cell lines, RaBV failed to replicate significantly in vertebrate cells when incubated at 37 °C with growth only apparent when incubated at 34 °C or below [45]. Suggestions that RaBV may have adapted to a transmission cycle without vertebrate hosts or used hosts with lower body temperatures, such as reptiles or amphibians are similarly applicable hypotheses for PLV. Future studies investigating the inability of PLV to infect vertebrate cells should consider temperature sensitivity as a possible restriction factor in its host tropism.

## 5. Conclusions

In summary, we have identified and characterised a new virus (PLV), belonging to the *Reoviridae* family. Analysis of highly conserved segments as well as serological cross-reactivity and neutralization analyses indicate that PLV belongs to the Corriparta serocomplex. However, in a fundamental difference to CORV, PLV failed to replicate in the vertebrate cell lines tested here, which may warrant its classification as a new species within the serocomplex. While these findings do suggest that PLV represents the first isolation and characterisation of an insect-specific member of the *Orbivirus* genus discovered in Australia, future *in vivo* and *in vitro* studies are warranted to confirm a mosquito-only life cycle. The discovery and characterisation of additional mosquito-associated orbiviruses will provide valuable insights into genetic divergence, extending and enhancing our understanding of the mosquito virome.

## Figures and Tables

**Table 1:** Summary of mosquito pools that tested positive for PLV by RT-PCR screening.

Pool ID	Mosquito Species	Date Collected	Initial Isolation			Re-Isolation	% Pairwise Identity to Prototype (nt/aa) *^e^*
			CPE	CPE	CPE	CPE	
			C6/36 *^a^*	Vero *^b^*	PS-EK *^b^*	C6/36 *^c^*	
Kununurra							
K74511	*Culex pullus*	April 2011	+	−	−	+	99.75/99.25
K74544	*Culex pullus*	April 2011	+	−	−	+	ND
K74770	*Culex annulirostris*	April 2011	+	−	−	+	99.75/99.25
K74775	*Culex species*	April 2011	+	−	−	+	99.75/99.25
K75023	*Culex annulirostris*	April 2011	+	−	−	+	99.75/99.25
Parry’s Creek							
K71497	*Culex annulirostris*	March 2010	−	−	−	+	97.47/95.49
K71516	*Culex annulirostris*	March 2010	−	−	−	−	97.74/95.49
K71520	*Culex annulirostris*	March 2010	−	−	−	−	97.74/95.49
**K71551**	***Culex annulirostris***	**March 2010**	−	−	−	+	**100/100**
K71558	*Culex annulirostris*	March 2010	−	−	−	−	97.74/95.49
K71435	*Culex annulirostris*	March 2010	−	−	−	+	99.50/98.50
K75533	*Mansonia uniformis*	April 2011	+	−	−	+	99.75/99.25
Billiluna							
K75737	*Aedes normanensis*	April 2011	+	−	−	+	99.75/99.25
K75749 *^d^*	*Culex annulirostris*	April 2011	+	+ *^d^*	+ *^d^*	+	ND

*^a^* On the 2nd passage; *^b^* 3rd passage (after 2 on C6/36 cells); *^c^* On the 3rd passage; *^d^* = indicative of a co-infection; *^e^* identity calculated over 399 bp region of segment 9; (+) CPE positive; (−) CPE negative; ND = Not Done.

**Table 2:** PLV Genome Organisation.

Segment	Protein Encoding	G+C Content (%)	ORF Length (bp)	Predicted Protein Length (aa)	% Pairwise Identity with CORV (nt/aa)
1	VP1 (Pol)	40.8	3873	1290	78.1/91.9
2	VP2 (T2)	41.8	2850	949	79.9/94.3
3	VP3 (OC1)	42.2	2217	738	74.7/85.8
4	NS1 (TuP)	48.0	1770	589	78.0/89.8
5	VP5 (OC2)	45.3	1584	527	79.1/95.3
6	VP4 (Cap)	44.4	1932	643	78.1/90.4
7	NS2 (ViP)	46.2	1116	371	81.1/92.7
8	VP7 (T13)	46.8	1065	352	79.2/94.3
9	VP6 (Hel)/NS4	46.0	1035	344	78.0/72.6
10	NS3/NS3a	46.7	717	238	81.2/92.0

Pol = Polymerase; OC1 = Outer capsid protein; T2 = Inner core protein (T2 symmetry); Cap = capping enzyme; TuP = tubule forming protein or tubular protein (NS1); OC2 = outer capsid protein 2; T13 = outer core protein (T13 symmetry); ViP = viral inclusion body protein (NS2); Hel = helicase protein.

**Table 3:** Pairwise amino acid identities between CORV, PLV and CMPV.

		Percent Identity *^a^*	
Protein	Virus	CORV	PLV
VP2	CMPV	92	87
	CORV		95
VP4	CMPV	70	71
	CORV		90
VP5	CMPV	83	84
	CORV		95
VP7	CMPV	92	94
	CORV		94
VP6	CMPV	39	42
	CORV		73
NS4	CMPV	56	58
	CORV		77

***^a^*** Alignments truncated according to available sequence for CMPV; CMPV—California Mosquito Pool virus; CORV—Corriparta virus MRM1; PLV—Parry’s Lagoon virus.

**Table 4:** Cross-neutralization of PLV with CORV antiserum.

Virus	CORV Antiserum 1 (B97)	CORV Antiserum 2 (CSIRO109)	Negative
CORV	80	320	<10
PLV	20	>1280	<10

## Supplementary Materials

The following are available online at www.mdpi.com/1999-4915/8/5/141/s1, Figure S1: Phylogenetic analysis of truncated *orbivirus* highly conserved T13 gene.
