# Supplementary material for: A New Orbivirus Isolated from Mosquitoes in North-Western Australia Shows Antigenic and Genetic Similarity to Corriparta Virus but Does Not Replicate in Vertebrate Cells
Source: Viruses. 2016 May 20;8(5):141. doi: 10.3390/v8050141 (PMC4885096; doi:10.3390/v8050141)
Supplement: Supplementary file 1 [file viruses-08-00141-s001.pdf]

Supplementary Materials: A New Orbivirus Isolated from Mosquitoes in North Western Australia Shows Antigenic and Genetic Similarity to Corriparta Virus But Does Not Replicate in Vertebrate Cells

Jessica J. Harrison, David Warrilow, Breeanna J. McLean, Daniel Watterson, Caitlin A. O'Brien, Agathe M.G. Colmant, Cheryl A. Johansen, Ross T. Barnard, Sonja Hall-Mendelin, Steven S. Davis, Roy A. Hall and Jody Hobson-Peters

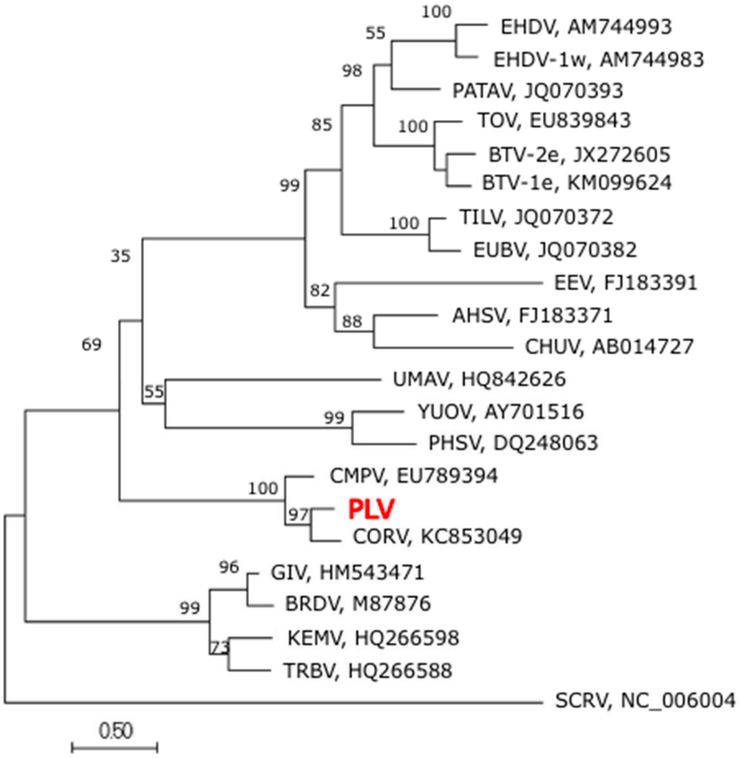

Figure S1. Phylogenetic analysis of truncated *orbivirus* highly conserved T13 gene. Maximum likelihood phylogenetic tree of highly conserved T13 (outer core) gene was constructed using a general time reversible model with gamma distribution and invariant sites (as determined by a jModelTest) with 1,000 replicates. "e" and "w" denotes Eastern and Western isolates respectively. Accession numbers are denoted next to each virus. PLV accession numbers: KU724110-KU724119.

© 2016 by the authors; licensee MDPI, Basel, Switzerland. This article is an open access article distributed under the terms and conditions of the Creative Commons by Attribution (CC-BY) license (http://creativecommons.org/licenses/by/4.0/).
